# Commentary on ‘Moral reasons to edit the human genome’: this is not the moral imperative we are looking for

**DOI:** 10.1136/medethics-2018-105316

**Published:** 2019-08-27

**Authors:** Sarah Chan

**Keywords:** ethics, genetic information, genetic engineering

After reading Savulescu and colleagues,^1^ one ought to be in no doubt that human heritable genome editing (HGE) is a ‘moral imperative’: to cure disease, reduce inequalities, improve public health and protect future generations. They make this argument repeatedly and in no uncertain terms. Yet are they right to do so?

I am certainly not against developing HGE or exploring its possibilities. Instead, I aim to sound a cautionary note in relation to claims about its technological potential and how we frame arguments on this basis.

The ‘moral imperative’ argument (MIA) has been made many times, since well before the advent of genome editing, by the present authors and others (eg, refs.^2–4^). It generally rests on a number of preconditions, implicit or explicit: that HGE will be safe, effective, cost-efficient and equitably available. Now, many bioethicists (myself included) would take no issue with, indeed have supported,^5^ the proposition that, *once* all of these conditions are satisfied, HGE is something we have good moral reasons to pursue. At this pivotal moment for global science, ethics and governance, however, we need equally to be concerned with the technology’s immediate future trajectory: *whether and how* we can reach the point of satisfying these conditions. In this context, the MIA is something of a distraction; at worst, it may even be corrosive and damaging.

Consider first the argument from evolutionary fitness, that HGE is morally required to counteract the supposed ‘genetic deterioration’ produced by medically enabled ‘survival of the weak’, that makes us increasingly ‘reliant on technology’.^1^ Of course, I would prefer not to have to wear glasses to correct my vision; this must be doubly so for more intrusive, life-limiting interventions. As modern humans, however, we are dependent on a whole host of technologies, from which genes may or may not emancipate us. Focusing on genes as the answer to society’s problems distracts from the more fundamental imperative of ensuring that *via whatever means*, people have access to the resources they require in order to thrive and participate in society. We should not neglect the social and environmental determinants of well-being in favour of pursuing the ‘moral imperative’ of genetic solutions.

To be fair, Savulescu and colleagues do not assert that genes are the only or best solution; they allow that ‘which intervention we ought to choose… depends on the costs and benefits of the particular intervention, and relevant moral values’. Nevertheless, the repeated framing of the argument around HGE as a ‘moral imperative’ (and what is thereby omitted from the discussion) implicitly centres genetics, pushing other considerations to the margins.

The authors further argue that modern medicine is ‘worsening the position of members of the future generation[s], by allowing random mutations to occur to our genome’. Yet (as they well know) enabling people to survive and reproduce cannot be said to worsen the position of their direct future descendants, who would not otherwise exist. Is the implication, then, that some individuals in future generations will be made worse off by allowing the existence of ‘genetically weaker’ contemporaries who will contribute less to, and require more resources from, future society? This seems a worrying approach to what collective responsibility requires, specifically that certain individuals not be (permitted to be) brought into existence. Bioethicists have taken pains to disentangle the literal meaning of ‘eugenics’, together with pro-HGE arguments based on respect for individual and collective welfare, from the dire sociopolitical connotations and human rights abuses historically associated with the term. Eliding individual and collective interests and responsibilities in this way threatens to re-entangle them, with potentially dangerous consequences.

This brings us to further concern. HGE might exacerbate social division and marginalisation not only via the use of technology itself, but also by the ethical, political and public discourse surrounding it: the hope, hype and imaginaries attached to the future of genome editing. As bioethicists, we must be conscious of how the arguments we advance, as well as when and how we choose to do so, affect this discourse.

The MIA, as articulated by Savulescu and colleagues, rests partly on parity between genetic and non-genetic, or heritable and non-heritable means of achieving a given desirable individual, societal or public health goal. If non-genetic means of attaining this goal are considered obligatory, they argue, genetic interventions that achieve the same aim are likewise a moral imperative; moreover, many non-genetic interventions will affect future generations and have lasting effects. In itself, this is a sound argument, but is it the one we most need now?

Previously, when both the genetic nature and heritability of interventions were widely perceived as intrinsic grounds for objection, the MIA played an important role in reframing the issues to challenge these assumptions. Today, however, the discourse has shifted: not only the Nuffield Council report^6^ but several other statements (reviewed in ref.^7^) have acknowledged HGE as potentially ethically acceptable. Under these circumstances, simply rearticulating the far-future-oriented, highly conditional MIA may not be the most useful response. Or, though perhaps it still bears saying, we should be careful how we say it: the benefits of presenting the argument in a certain form should justify potential negative consequences.

In this respect, the MIA in relation to intelligence is perhaps most concerning. Although we currently understand and might be able to address the genetic basis of a very few forms of severe cognitive impairment, using HGE to improve cognitive ability more generally is a vastly different prospect. Given this far-fetched, uncertain payoff, plus the history of coercive practices in relation to cognitive impairment and genetics, framing the MIA in this way seems a poor choice.

First, it ignores the social context in which the argument will inevitably be read and in which it is liable to reinforce the very genetic essentialism and exceptionalism that it tries to counteract. Further, it may actually hinder the development of HGE, through polarising the debate and stirring up the negative sentiment. Witness for example the controversy resulting from recent proposals for ‘genetically-sensitive education’^8 9^; the idea that differences in achievement are a genetic problem in need of a genetic solution is similarly likely to provoke ‘hostility and misconceptions’,^8^ impeding progress towards realising the ‘moral imperative’.

Finally, one cannot ignore the probability of this argument being co-opted to justify discrimination on the basis of pseudo-genetics. Pinning disadvantage on genetic factors while HGE-mediated interventions remain out of reach may not only distract from current solutions but entrench prejudice and marginalisation. HGE’s most immediate threat is not its direct use in the future to bring about some dystopian vision of GenRich versus GenPoor. Rather, it is the indirect effect of quasi-utopian discourse over potential future uses that, in overstepping current understandings and limitations, inadvertently promotes geneticisation in the present. For the sake of bioethical, as well as scientific, responsibility, we must avoid this.

## References

[1] Gyngell C, Bowman-Smart H, Savulescu J Moral reasons to edit the human genome: picking up from the Nuffield report. J Med Ethics 2019;45:513–22. 10.1136/medethics-2018-105084 PMC6820147 30679191

[2] Harris J Wonderwoman and superman: the ethics of human biotechnology. Oxford: Oxford University Press, 1992.

[3] Savulescu J New breeds of humans: the moral obligation to enhance. Reprod Biomed Online 2005;10(Suppl 1):36–9. 10.1016/S1472-6483(10)62202-X 15820005

[4] Savulescu J, Pugh J, Douglas T, et al The moral imperative to continue gene editing research on human embryos. Protein Cell 2015;6:476–9. 10.1007/s13238-015-0184-y 26113289 PMC4491050

[5] Chan S, Harris J The ethics of gene therapy. Curr Opin Mol Ther 2006;8:377–83. 17078379

[6] Nuffield Council on Bioethics. Genome editing and human reproduction. London, 2018.

[7] Brokowski C Do CRISPR germline ethics statements cut it? Crispr J 2018;1:115–25. 10.1089/crispr.2017.0024 31021208 PMC6694771

[8] Crosswaite M, Asbury K ’Mr Cummings clearly does not understand the science of genetics and should maybe go back to school on the subject': an exploratory content analysis of the online comments beneath a controversial news story. Life Sci Soc Policy 2016;12:11 10.1186/s40504-016-0044-4 27812855 PMC5095087

[9] Tabery J Why is studying the genetics of intelligence so controversial? Hastings Cent Rep 2015;45(5 Suppl):S9–S14. 10.1002/hast.492 26413953

